# Short-Term Effect of Ozone Exposure on Small Airway Function in Adult Asthma Patients with PM_2.5_ Exacerbating the Effect

**DOI:** 10.3390/toxics13040279

**Published:** 2025-04-05

**Authors:** Ying Shang, Yanjing Liang, Dongxia Jiang, Zhengxiong Li, Xianlin Mu, Xuehu Han, Xinzhuo Xie, Guanglong Fu, Yunshu Zhang, Yongchang Sun, Shaodan Huang, Chun Chang

**Affiliations:** 1 Department of Respiratory and Critical Care Medicine, Peking University Third Hospital, Beijing 100191, China; 2 Research Center for Chronic Airway Diseases, Peking University Health Science Center, Beijing 100191, China; 3 Department of Occupational and Environmental Health Sciences, School of Public Health, Peking University, Beijing 100191, China; 4 School of Public Health, Peking University, Beijing 100191, China; 5 The Third School of Clinical Medicine, Peking University, Beijing 100191, China; 6 Key Laboratory of Epidemiology of Major Diseases, Peking University, Ministry of Education, Beijing 100191, China

**Keywords:** asthma, small airway function, ambient pollution, ozone (O_3_), fine particulate matter (PM_2.5_)

## Abstract

Ambient ozone (O_3_) has been associated with asthma symptoms and exacerbations. The impairment of small airway function leads to worse control, more frequent exacerbations and increased bronchial hyperresponsiveness in asthma patients. However, the impact of O_3_ on small airway function in asthma remains underexplored. Our longitudinal observational study enrolled 312 adult asthma patients and collected a total of 399 lung function records. We applied a linear mixed-effects model to analyze the associations between ambient O_3_ exposure at different lag days (from lag0 to lag7) and small airway function parameters, including forced expiratory flow (FEF) at 50%, 75% and 25–75% of forced vital capacity (FVC) predicted (FEF50%pred, FEF75%pred and FEF25–75%pred). Significant associations were found between ambient O_3_ levels and reductions in FEF50%pred, FEF75%pred and FEF25–75%pred, with the effects being most pronounced for exposure at lag0. Further analysis indicated that fine particulate matter (PM_2.5_) and its main components, including black carbon, organic matter, sulfate, nitrate and ammonium, exacerbated the detrimental effects of O_3_ on small airway function. Additionally, stronger O_3_ effects were found in asthma patients aged over 40 years, those with a body mass index ≥ 25 kg/m^2^, and individuals with allergic asthma. These results provide new insights into the impact of O_3_ on small airway function, offering fresh insights into asthma exacerbation mechanisms and underscoring the critical need to address composite pollutants for more effective asthma management.

## 1. Introduction

Asthma is a widespread chronic respiratory disease that is accompanied by variable expiratory airflow limitations and airway hyperresponsiveness, manifesting as symptoms including wheezing, dyspnea, chest constriction, and coughing. Globally, asthma affects over 260 million individuals, with prevalence rates ranging from 1% to 29% across different countries [1,2]. In China, approximately 45.7 million adults suffer from asthma, with uncontrolled symptoms and acute exacerbations leading to a decreased quality of life and a substantial healthcare burden [3]. Air pollution and meteorological factors are recognized as significant risk factors for asthma, contributing to an increased likelihood of exacerbations and hospitalizations [4]. In addition to standard treatment approaches, identifying and avoiding environmental risk factors are crucial for effective asthma management.

Ozone (O_3_) is a significant ambient pollutant primarily produced through photochemical reactions involving volatile organic compounds (VOCs) and nitrogen oxides (NOx), influenced by heat, sunlight, ultraviolet radiation and humidity, as well as by other air pollutants like particulate matter [5]. It is estimated that annual asthma-related emergency room visits attributed to O_3_ range from 9 to 23 million worldwide, accounting for 8–20% of all such visits [6]. Short-term exposure to O_3_ exacerbates respiratory symptoms, heightens the risk of emergency room visits and hospitalizations due to asthma exacerbation, and even increases asthma-related mortality rates [4,7].

The lung function test is among the most objective measures for assessing asthma severity, control and progression. Short- and long-term exposure to O_3_ can adversely affect lung function, with asthma patients demonstrating greater susceptibility to its detrimental effects than healthy individuals [8,9,10]. However, studies on the impacts of O_3_ exposure on lung function have focused mainly on proximal large airway obstruction parameters, e.g., forced expiratory volume in one second (FEV1) and the ratio of FEV1 to forced vital capacity (FEV1/FVC), reductions in which usually reflect structural changes in the airways during advanced stages and may indicate chronic irreversible airflow limitation.

The impairment of small airways, characterized by a diameter of less than 2 mm in the peripheral airways, occurs from the early stage of asthma and is considered a precursor to proximal airway dysfunction [11]. Small airway dysfunction (SAD) is associated with asthma across all severity stages, with an increasing prevalence corresponding to the intensity of treatment steps, and is associated with poorer disease control, more frequent exacerbations, and heightened bronchial hyperresponsiveness, which has led to greater clinical focus in recent years [12,13]. Forced expiratory flow (FEF) at 50%, 75% and 25–75% of FVC (FEF50%, FEF75% and FEF25–75%) are reliable spirometry parameters for assessing small airway function [14]. Owing to its low water solubility, O_3_ can penetrate into distal regions, including the terminal bronchioles, the junctions of bronchioles and alveolar ducts, and the proximal alveolar zones [15]. Prediction models for SAD have identified O_3_ exposure as a significant risk factor [16]. The China Pulmonary Health (CPH) study reported that long-term O_3_ exposure is associated with impaired small airway function and increased SAD risk [17]. However, existing studies typically involve the general population, and knowledge about the short-term effects of O_3_ on small airways in patients with asthma is very limited. Therefore, in this study, we established an asthma cohort to investigate the short-term effects of O_3_ exposure and its interaction factors on small airway function, with the aim of enhancing our understanding of the impact of O_3_ exposure on adults with asthma and thereby improving management strategies.

## 2. Methods

### 2.1. Study Population

We enrolled 312 asthma patients who presented at Peking University Third Hospital from May 2021 to July 2023. Repeated lung function measurements were taken during this period, and all lung function records of the participants were collected. All participants had physician-diagnosed asthma on the basis of the Global Initiative for Asthma 2021 guidelines [18]. Allergic asthma was recognized when asthma symptoms were triggered or worsened by exposure to aeroallergens, accompanied by a positive result in at least one serum test for aeroallergen-specific immunoglobulin E (IgE). Patients with any other respiratory disease, including chronic obstructive pulmonary disease, bronchiectasis, pneumonia, obstructive sleep apnea-hypopnea syndrome, malignant tumors, acute or chronic respiratory failure, serious cardiovascular disease, and other conditions that may affect ventilation function such as neurological and thorax diseases were excluded. Demographic information including age, sex, body mass index (BMI), smoking state (current smoker, ex-smoker, non-smoker), and residential address was collected at baseline, and medication information was obtained on the day of lung function testing. The recruitment of asthma patients and collection of demographic and clinical data were approved by the Ethics Committee of Peking University Third Hospital (approval numbers M2021185 and M2022148).

### 2.2. Lung Function Tests

In this study, lung function was assessed via standard spirometry (Elite series, MGC Diagnostics, St. Paul, MN, USA) by experienced operators. The participants were instructed to inhale and exhale, and the duration of each breath should be at least six seconds, with acceptable flow-volume variation. The measurements were repeated three times to obtain the best measurements for analysis. The spirometric parameters FEF50%pred, FEF75%pred, and FEF25–75%pred before and after the administration of bronchodilators were recorded.

### 2.3. Environmental Exposures

In this study, ambient air pollutants including O_3_, fine particulate matter (PM_2.5_) and its main constituents including black carbon (BC), organic matter (OM), sulfate (SO_4_^2−^), nitrate (NO_3_^−^) and ammonium (NH_4_^+^) were sourced from the China Tracking Air Pollution (TAP) dataset. Maximum daily average 8 h O_3_ levels and daily average PM_2.5_ concentrations were acquired at a resolution of 10 km × 10 km [19]. The daily temperature (T) and relative humidity (RH) data were obtained from the fifth generation of the European Reanalysis (ERA5)-Land Reanalysis (RLR) dataset provided by the European Center for Medium Range Forecasts (ECMWF), with a resolution of 9 km × 9 km [20]. We then used an application programming interface provided by the Amap to geocode the residential address of the subjects into latitude and longitude data. By aligning geographic coordinates with corresponding dates, a spatiotemporal correlation was established between atmospheric pollutant concentrations, meteorological factors, and small airway function parameters.

### 2.4. Statistical Analyses

This study employed a linear mixed-effects (LME) model to assess the lagged impacts of ambient O_3_ on small airway function. Statistical analyses were performed using R 4.2.2 software with the package “lme4”. The inclusion of a random intercept for each participant in the LME model accounted for individual-level variability in repeated measurements. Lag0 is the day on which the lung function test is performed, and lag1 is the day before the test. To investigate potential delayed impacts, we established a maximum lag period of 7 days, while adjusting for concurrent meteorological variables, such as temperature (lag0 T) and relative humidity (lag0 RH). Additionally, the model included adjustments for age, gender, BMI, smoking history, allergic status, and medication usage.

We conducted stratified analyses to investigate the interactions of O_3_ with PM_2.5_ and T on lung function. The subjects were divided into subgroups based on their exposure levels to ambient PM_2.5_ and its primary components (≥median or <median) and T levels (≥median or <median). The warm season was defined as the period from April to September, while the cold season spans from October to March of the following year. Subsequently, comparisons were conducted to assess the effects of O_3_ on small airway function across these two stratified groups.

We performed subgroup analyses on the basis of age (>40 or ≤40 years old), sex (female or male) and BMI (≥25 or <25 kg/m^2^) to explore potential modifying effects. To explore the effects of medication and allergic status, participants were categorized on the basis of whether they were receiving treatments such as inhaled or systemic corticosteroids and biologics, as well as their classification into allergic asthma subtypes.

To assess the robustness of our statistical models, we performed sensitivity analyses. We incorporated PM_2.5_ concentration as a control variable in our model to mitigate its influence on the effect of O_3_ on small airway function. Additionally, we utilized the lag0–7 moving average of T and RH to account for potential delayed effects of meteorological factors. The coefficients and 95% confidence intervals (CIs) of small airway function parameters associated with O_3_ were determined in all tests and represented changes in small airway parameters with a per 1 μg/m^3^ increase in ambient O_3_. A two-tailed value of *p* < 0.05 was considered statistically significant.

## 3. Results

### 3.1. Study Population and Environmental Variables

**Figure 1:**
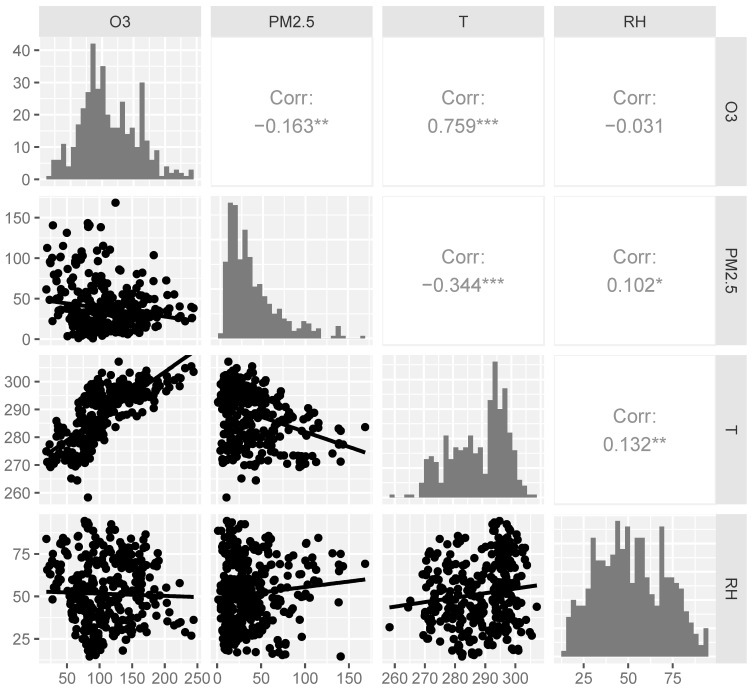
Spearman’s correlation coefficients for air pollutant concentrations and meteorological parameters at lag0 (*n* = 399). * *p* < 0.05, ** *p* < 0.01, *** *p* < 0.001.

This panel study included 312 asthma patients with a total of 399 lung function records. Table 1 presents the baseline demographic and small airway function information of the cohort. A total of 55.1% of the cohort were female, and 91.0% reported never smoking. The mean age was 40.87 years, with an average BMI of 24.26 kg/m^2^. Prebronchodilator (pre-BD) measurements for FEF50%pred, FEF75%pred, and FEF25–75%pred averaged 71.74%, 61.96% and 70.71%, respectively. The postbronchodilator (post-BD) values were slightly higher: 79.57%, 70.93%, and 78.86%, respectively. The ambient environmental variable information of the day the lung function tests were conducted is summarized in Table 2. The average concentrations of O_3_ and PM_2.5_ were 111.01 μg/m^3^ and 37.49 μg/m^3^, respectively. There are 272 instances in the warm season where the average O_3_ concentration reached 129.72 μg/m^3^, significantly higher than in the cold season (*p* < 0.001). Additionally, the PM_2.5_ concentration in the cold season was significantly higher than in the warm season (*p* < 0.001). The average temperature was 288.71 K, with an RH of 51.54%. We analyzed the correlations among the ambient environmental variables via Spearman correlation analyses. Notably, O_3_ showed a strong positive correlation with temperature (r = 0.759, *p* < 0.001) and a weak negative correlation with PM_2.5_ (r = −0.163, *p* < 0.01), as illustrated in Figure 1.

### 3.2. Effects of Ambient O_3_ on Small Airway Function in Patients with Asthma

**Figure 2:**
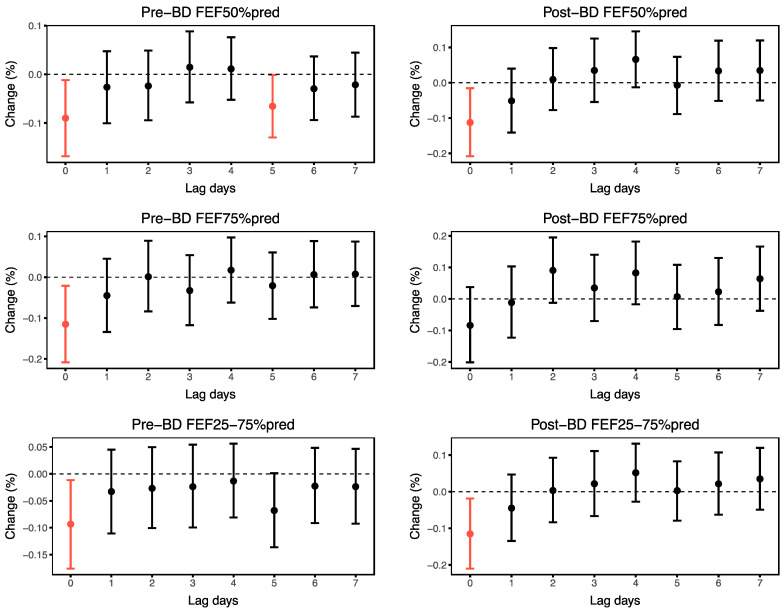
Associations between small airway function parameters and ambient O_3_ at lag0–lag7. The graphs show changes in small airway function parameters per 1 μg/m^3^ increase in O_3_ exposure. The red lines indicate *p* < 0.05.

The associations between ambient O_3_ and small airway function parameters in the asthma cohort are shown in Figure 2. Ambient O_3_ levels were significantly associated with FEF50%, FEF75% and FEF25–75%. The most pronounced association was observed at lag0. Specifically, a 1 μg/m^3^ increase in O_3_ at lag0 was associated with a 0.09% (95% CI: −0.17, −0.01) decrease in pre-BD FEF50%pred, a 0.11% (95% CI: −0.21, −0.02) decrease in post-BD FEF50%pred, a 0.12% (95% CI: −0.21, −0.02) decrease in pre-BD FEF75%pred, a 0.09% (95% CI: −0.18, −0.01) decrease in pre-BD FEF25–75%pred and a 0.12% (95% CI: −0.21, −0.02) decrease in post-BD FEF25–75%pred.

### 3.3. Interactions of Ambient O_3_ with PM_2.5_

**Figure 3:**
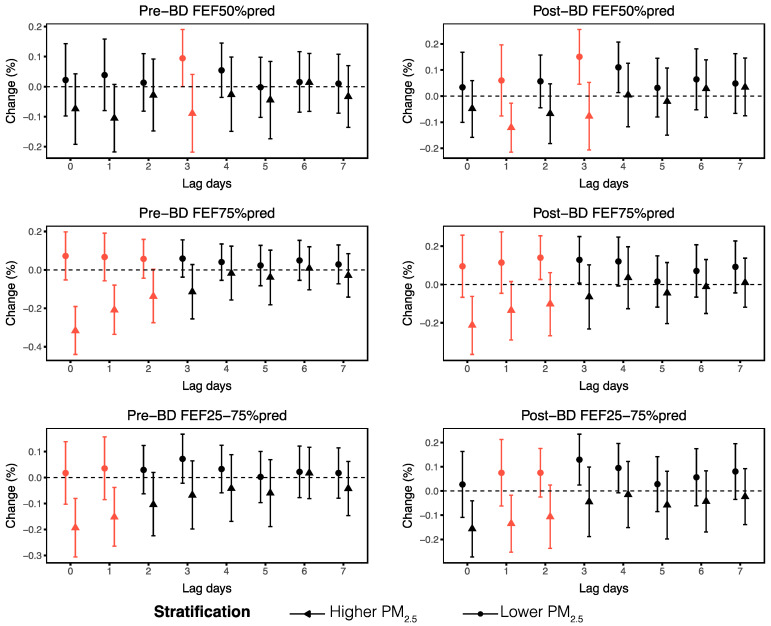
Effects of ambient O_3_ at lag0–lag7 on small airway function parameters across different PM_2.5_ levels. The red lines indicate significant differences in the associations between O_3_ and small airway function parameters at higher and lower PM_2.5_ levels (*p* < 0.05).

We subsequently analyzed the associations between O_3_ and small airway function parameters across different levels of PM_2.5_. Significant variations were observed in the changes of FEF50%pred, FEF75%pred, and FEF25–75%pred in response to a 1 μg/m^3^ increment in O_3_ exposure across varying levels of PM_2.5_ concentration, particularly at lag0–3. Higher PM_2.5_ levels notably exacerbated the negative effects of O_3_ on small airway function over the short term (Figure 3). To assess the independent effect of PM_2.5_, we analyzed the associations between PM_2.5_ and small airway function parameters. However, no significant correlation was found over short time lags (Figure S1). These findings indicate that O_3_ interacts with PM_2.5_ to affect small airway function.

**Figure 4:**
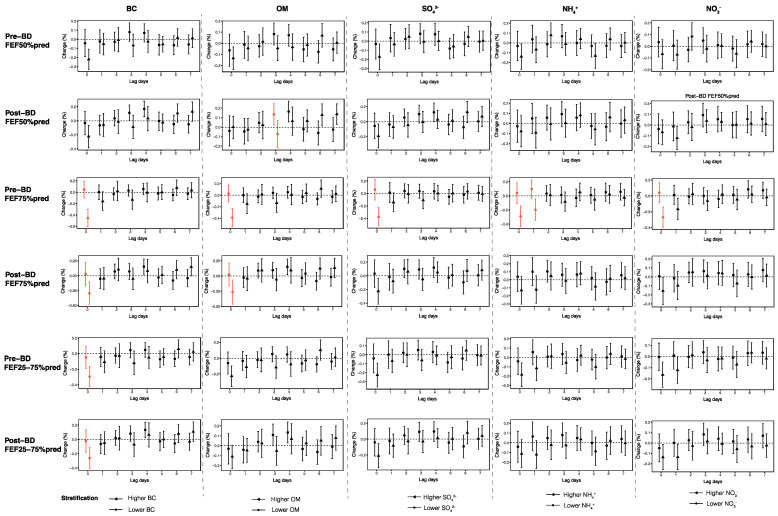
Effects of ambient O_3_ at lag0–lag7 on small airway function parameters across different levels of PM_2.5_ constituents. The red lines indicate significant differences in the associations between O_3_ and small airway function parameters at higher and lower levels of PM_2.5_ constituents (*p* < 0.05).

PM_2.5_ is composed of carbonaceous components and water-soluble ions, exhibiting varying toxicity to human health [21]. In this study, we further investigate the contributions of PM_2.5_ five main constituents (BC, OM, SO_4_^2−^, NO_3_^−^, and NH_4_^+^) to the effects of O_3_ on small airway function. Notable variations were detected in the changes of pre-BD FEF75%pred at lag0, following a 1 μg/m^3^ increment in O_3_ exposure, across varying concentrations of the five primary components (Figure 4). Specifically, BC, OM and SO_4_^2−^ were found to have a larger effect on the relationship between O_3_ and pre-BD FEF75%pred. Moreover, BC and OM appear to play particularly significant roles. In environments with higher BC levels, post-BD FEF75%pred, pre- and post-BD FEF25–75%pred at lag0 also exhibited a more pronounced decrease with each 1 μg/m^3^ increase in O_3_. Similarly, in environments with elevated OM levels, post-BD FEF50%pred at lag3 and post-BD FEF75%pred at lag0 also showed greater reductions with each 1 μg/m^3^ increase in O_3_ (Figure 4).

### 3.4. Stratified Analyses

O_3_ levels are generally higher during warmer seasons, which aligns with our observations of a strong correlation between O_3_ concentrations and temperature. Consequently, we further explored the relationship between O_3_ and small airway function across varying temperature levels (Figure S2). However, we found no significant variation in the decline of small airway function with each 1 µg/m^3^ increase in O_3_ concentration across different temperature levels. Similarly, stratified analysis by season revealed no significant difference in the associations between O_3_ and small airway function parameters between warm and cold seasons (Figure S3).

**Figure 5:**
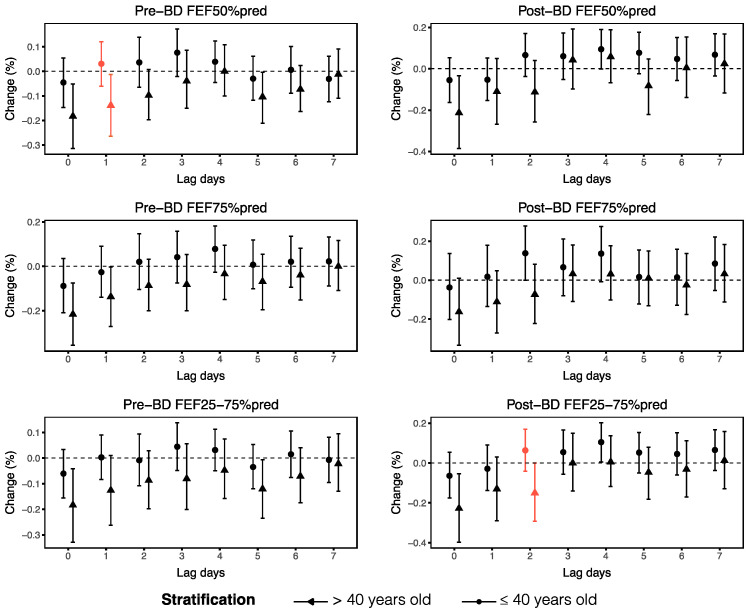
Stratified analyses of the associations between ambient O_3_ at lag0–lag7 and small airway function parameters on the basis of age. The red lines indicate significant differences in the associations between O_3_ and small airway function parameters in individuals over and under 40 years of age (*p* < 0.05).

**Figure 6:**
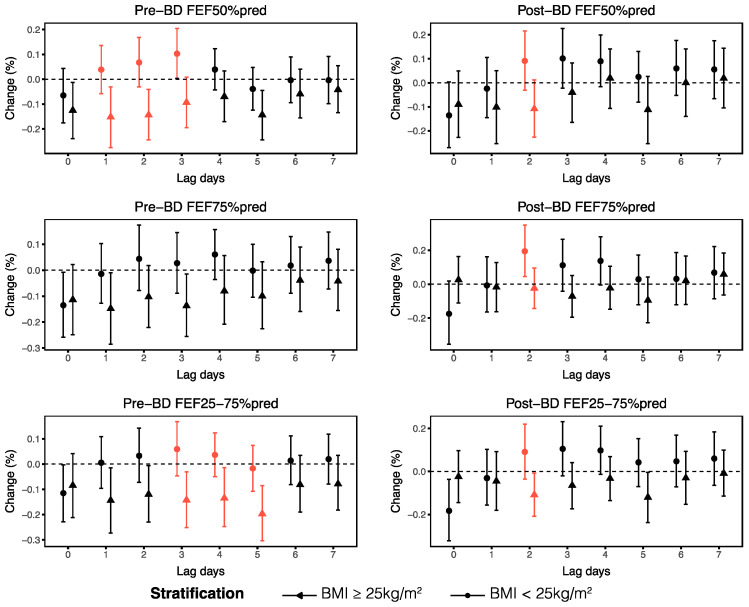
Stratified analyses of the associations between ambient O_3_ at lag0–lag7 and small airway function parameters on the basis of BMI. The red lines indicate significant differences in the associations between O_3_ and small airway function parameters in individuals with BMI greater than 25 kg/m^2^ and less than 25 kg/m^2^ (*p* < 0.05).

We then performed stratified analyses to evaluate the effects of O_3_ on small airway function across demographic and clinical factors, including age, sex, BMI, allergic asthma subtypes and medication use. Our findings revealed that asthma patients over the age of 40 years and those with a BMI of 25 kg/m^2^ or higher experienced more serious impairments in small airway function parameters when exposed to O_3_. Specifically, we observed that pre-BD FEF50%pred and post-BD FEF25–75%pred decreased more significantly with each 1 μg/m^3^ increase in O_3_ in patients aged over 40 years, with changes of −0.14% (95% CI: −0.26, −0.01) at lag1 and −0.15% (95% CI: −0.29, 0.00) at lag2, respectively (Figure 5). According to the BMI-stratified analyses, FEF50%, FEF75% and FEF25–75% were more susceptible to O_3_ in asthma patients with higher BMIs, as indicated by significant differences across different BMIs at different lag days (Figure 6). Additionally, our analyses did not reveal significant differences in the associations between small airway function and ambient O_3_ from lag0 to lag7 among the different sex groups (Figure S4).

Asthma is a heterogeneous disease, with allergic asthma being the most prevalent phenotype characterized by sensitization to aeroallergens and accompanied by Th2 inflammation [22]. Our subgroup analyses revealed that small airway function indices in allergic asthma patients may be more susceptible to O_3_ exposure, as post-BD FEF25–75%pred tends to decrease more significantly in allergic asthma patients, with a change of −0.09% (95% CI: −0.20, 0.03) per 1 μg/m^3^ increase in O_3_ at lag1 (Figure S5). To explore the potential effects of medication use, we conducted a stratified analysis according to whether patients were receiving treatment with inhaled or systemic corticosteroids and biologics. The results indicated that there were no significant differences in the effects of O_3_ on small airway function within lag0–3 regardless of medication use, despite post-BD FEF75%pred and pre-BD FEF25–75%pred decreasing significantly more in patients receiving regular medication when exposed to O_3_ at lag6 (Figure S6).

### 3.5. Sensitivity Analyses

For sensitivity analyses, we further incorporated PM_2.5_ concentration as a control variable in our model. The relationships between ambient O_3_ levels and small airway function parameters were hardly affected (Figure S7). Additionally, we substituted lag0 T and RH with their averages spanning from lag0 to lag7. The trends observed in the associations between ambient O_3_ and small airway function indicators remained consistent (Figure S8).

## 4. Discussion

In this study, we evaluated the short-term effects of ambient O_3_ exposure on small airway function in asthma patients. We identified significant associations between short-term ambient O_3_ exposure and reduced small airway function parameters, including FEF50%, FEF75% and FEF25–75% in asthma patients. Elevated PM_2.5_ and its main constituents, BC, OM, SO_4_^2−^, NO_3_^−^ and NH_4_^+^, exacerbated the detrimental impacts of O_3_ on small airways. Notably, stronger negative correlations between O_3_ and small airway function indices were observed in asthma patients aged over 40 years, those with higher BMIs and those with an allergic asthma phenotype.

To our knowledge, this study represents an initial investigation into the short-term effects of O_3_ on small airway function within a cohort of adult asthma patients. The CPH study demonstrated that long-term O_3_ exposure can impair small airway function, with each 4.9 ppb increase in warm-season O_3_ concentration being associated with a 14.2 mL/s reduction in FEF75% and a 29.5 mL/s reduction in FEF25–75% [17]. Previous studies with smaller sample sizes have indicated that long-term exposure to environmental O_3_ is correlated with decreased levels of FEF75% and FEF25–75% in college students [23,24]. These suggest that chronic O_3_ exposure may cause irreversible damage to the small airways. Regarding the short-term impacts of O_3_, two studies conducted in Taiwan have reported that 1-day lagged O_3_ levels exhibited an inverse relationship with FEF50%, FEF75%, and FEF25–75% in schoolchildren [25,26]. Conversely, a study conducted in France failed to demonstrate any correlation between short-term exposure to O_3_ and parameters indicative of small airway function among non-smoking, healthy adults [27]. The inconsistency in the conclusions may be attributed to variations in spatiotemporal factors and population characteristics.

In our study, the strongest effect of O_3_ exposure was observed at lag0 rather than at later time points. O_3_ concentration exhibits rapid fluctuations. Exposure to O_3_ can lead to rapid disruption of the epithelial lining, followed by the recruitment of inflammatory cells and increased oxidative stress, resulting in increased permeability and tissue damage [28]. Short-term exposure to O_3_ can induce respiratory symptoms, precipitate asthma exacerbations, and even elevate asthma-related mortality rates [28]. Acute O_3_ exposure for 130 min can reduce FEF25–75% with the effect occurring at 25 min and persisting for 24 h in adults [29]. Arjomandi et al. reported the greatest decreases in FEF25–75% and FEF75% after asthma patients were exposed to O_3_ for 1–2 days, with effects gradually diminishing over subsequent days of multiday exposure [30]. Similarly, Jang et al. reported that increased airway responsiveness induced by short-term O_3_ did not persist during chronic exposure [31]. These findings align with our observation that FEF50%, FEF75% and FEF25–75% were associated with the O_3_ concentration at lag0, with correlations becoming nearly nonsignificant at longer time lags. One explanation could be airway remodeling and adaptation during repeated O_3_ exposure, although irreversible structural changes and emphysema may occur over time.

We did not find a significant association between PM_2.5_ levels and small airway function indices. However, we observed a stronger association between O_3_ and small airway function indices at higher PM_2.5_ levels, confirming the interaction effect of O_3_ and PM_2.5_ on small airway function. The short-term effects of PM_2.5_ exposure on small airway function remain controversial. A panel study reported the detrimental impacts of PM_2.5_ exposure on FEF25–75%, FEF50% and FEF75%, with the strongest effects observed at a 2 h moving average, persisting for 24 h [32]. In contrast, another longitudinal panel study revealed decreased FEF25–75% after PM_2.5_ exposure in patients with chronic obstructive pulmonary disease rather than asthma [33]. Additionally, an SAD prediction model revealed no association between small airway disorders and PM_2.5_ exposure [34]. From a long-term exposure perspective, the CPH study found that PM_2.5_ and its main components (OM, BC, SO_4_^2−^, NO_3_^−^, NH_4_^+^) exhibited significant inverse correlations with FEF25–75%, with OM and NO_3_^−^ showing stronger associations [35]. These discrepancies may arise from variations in population characteristics, regional variations, and cumulative effects. Additionally, higher PM_2.5_ concentrations are often more noticeable to individuals than O_3_, which may lead to reduced outdoor activity during periods of high PM_2.5_ levels, thus lowering overall exposure. With stricter regulations, current PM_2.5_ levels in the region may not be sufficient enough to produce noticeable independent effects. However, the interaction between O_3_ and PM_2.5_ is acknowledged, though the exact mechanisms remain unclear. The soluble components of PM_2.5_, primarily small ions, can damage cell viability through ROS-induced oxidative stress. In contrast, the insoluble components may induce cell membrane rupture via particle-cell interactions, both exacerbating airway damage induced by O_3_ [36]. Research on the interaction between O_3_ and ultrafine carbon particulates has shown that co-exposure to both aerosols increases acellular and cellular oxidants, leading to greater declines in respiratory function compared to individual exposures [37]. Consistent with our findings, a previous study has demonstrated that short-term O_3_ exposure is associated with respiratory mortality, while short-term PM_2.5_ exposure does not show a significant correlation. However, the combined effects of PM_2.5_ and O_3_ on respiratory mortality have been shown to exceed their individual effects [38]. Overall, the cumulative effects of multiple pollutants on respiratory health warrant greater emphasis.

Higher temperatures contribute to increased O_3_ production, potentially intensifying the association between asthma exacerbations and O_3_ during warmer seasons [39]. Extreme temperatures are linked to increased risks of asthma exacerbation and reduced lung function [40]. However, our analysis did not reveal any significant differences in the detrimental effects of O_3_ on small airway function between higher and lower temperature groups or across warm and cold seasons. This suggests that a 1 µg/m^3^ increase in O_3_ concentration results in a similar decline in small airway function, regardless of temperature levels or seasons. Previous research has shown that the decline in lung function following 0.3 ppm O_3_ exposure is comparable across moderate and elevated temperatures, which partly supports our findings [41]. Nevertheless, higher absolute concentrations of O_3_ are expected to lead to more pronounced declines in small airway function.

Our stratified analyses revealed that the small airway function of elderly patients and those with higher BMIs was more vulnerable to O_3_ exposure, which aligns with the results of previous studies. A national cross-sectional study in China revealed that SAD is more prevalent among elderly individuals and that increased BMI significantly increases the risk of SAD [14]. A predictive model for SAD has identified age as a risk factor [34]. Obesity is widely acknowledged to serve as a predisposing factor for asthma, and studies have reported that obese individuals experience enhanced airway responsiveness, greater decreases in lung function and more severe asthmatic symptoms due to O_3_ exposure [42]. Overall, our results imply that greater emphasis should be placed on elderly and obese asthma patients in light of the threat posed by O_3_.

The acute effects of ambient O_3_ on small airway function may vary depending on asthma phenotypes. We found that FEF25–75% and FEF75% were more vulnerable in allergic asthmatics when exposed to ambient O_3_ than in non-allergic asthmatics, which is consistent with previous research suggesting a lower risk of O_3_-associated effects in non-allergic asthma [43]. Studies have indicated that asthmatics with allergic comorbidities are more susceptible to ambient O_3_ for acute exacerbation, and susceptibility to antigen challenge can be increased by ambient O_3_ exposure in asthmatics [44]. In allergic asthma mouse models, O_3_ exposure exacerbates airway hyperresponsiveness, airway resistance, pulmonary inflammation and mucus production [45]. However, there have been limited investigations into the distinct impacts of O_3_ on allergic versus non-allergic asthma. Asthma treatments are considered to reduce inflammation and improve lung function. Inhaled corticosteroids have been shown in previous studies to mitigate the adverse effects of O_3_ on FEF25–75% in asthmatic children [46]. However, we did not observe a significantly increased risk among asthma patients who were not receiving treatment. The variability in asthma severity and symptom control may contribute to this, as patients undergoing treatment typically have greater therapeutic demands, and individuals with poor symptom control may exhibit increased susceptibility to O_3_ exposure. Generally, the impact of O_3_ pollution on asthma patients should not be considered lightly, irrespective of whether they are receiving regular medication.

There are several limitations to our study. Firstly, we used ambient O_3_ and PM_2.5_ exposures, which may not accurately reflect individual exposure levels. Future studies will focus on individual-level assessments, utilizing personal samplers or wearable devices to improve exposure accuracy and asking participants to maintain activity diaries documenting daily routines, commuting routes, and outdoor exposure to investigate the impact of air pollutants on pulmonary function in asthma patients more precisely. Secondly, while we primarily examined the interaction between PM_2.5_ and O_3_ on small airway function, we did not consider other pollutants, such as airborne pollen, dust mites, VOCs, and NOx, due to a lack of data, which may also influence the relationship between O_3_ and small airway function. Additionally, while we used lung function parameters, which are widely used in clinical practice and have been identified as predictors of small airway dysfunction, pathological examinations and imaging techniques provide a more precise assessment.

Nevertheless, our research offers unique advantages. We analyzed various small airway function-related indices (FEF50%, FEF75% and FEF25–75%) in asthma patients. Our study contributes to filling a research gap regarding the short-term impacts of O_3_ exposure on small airway function in asthmatic individuals, an area that has been previously underexplored. We also explored the interactions between O_3_ and other environmental factors, with a particular emphasis on the synergistic effects with ambient PM_2.5_ and its constituents. Furthermore, we identified specific characteristics of asthma patients who demonstrate increased susceptibility to O_3_ exposure.

## 5. Conclusions

This study revealed a short-term adverse impact of ambient O_3_ on small airway function of 312 adult asthma patients. We also observed that the adverse effects of O_3_ are exacerbated by PM_2.5_ and its constituents, especially BC and OM. Moreover, individuals over 40 years of age, those with a BMI ≥ 25 kg/m^2^, and those with allergic asthma are at an increased susceptibility to O_3_ exposure. Our findings offer fresh perspectives on the relationship between O_3_ and small airway function, emphasizing the necessity of addressing combined air pollutants to enhance asthma management and mitigate their detrimental impacts.

## Figures and Tables

**Table 1:** Baseline demographic and clinical characteristics of asthma patients (*n* = 312).

Descriptive Information of 312 Participates		
Variables		N (%)
Age (years)		
	Mean (SD)	40.87 (14.78)
	>40	136 (43.6)
	≤40	176 (56.4)
BMI (kg/m^2^) ^1^	Mean (SD)	24.26 (3.70)
	≥25	120 (38.5)
	<25	192 (61.5)
Sex		
	Female	172 (55.1)
	Male	140 (44.9)
Smoking status		
	Ex-smoker	8 (2.6)
	Current smoker	20 (6.4)
	Non-smoker	284 (91.0)
Allergic asthma ^2^		
	Yes	154 (49.4)
	No	114 (36.5)
Medication use		
	Yes	149 (47.8)
	No	163 (52.2)
**Small airway function parameters ^3^**	**Mean (SD)**	**Median (IQR)**
Pre-BD FEF50%pred (%)	71.74 (27.00)	70.20 (38.80)
Pre-BD FEF75%pred (%)	61.96 (28.08)	59.15 (34.83)
Pre-BD FEF25–75%pred (%)	70.71 (26.43)	69.85 (38.15)
Post-BD FEF50%pred (%)	79.57 (27.34)	77.15 (35.00)
Post-BD FEF75%pred (%)	70.93 (30.34)	68.00 (42.40)
Post-BD FEF25–75%pred (%)	78.86 (26.65)	76.80 (37.15)

^1^ BMI, body mass index; ^2^ forty-four asthma patients lacked aeroallergen tests; ^3^ pre-BD, prebronchodilator; post-BD, postbronchodilator; FEF50%pred, forced expiratory flow at 50% of forced vital capacity predicted; FEF75%pred, forced expiratory flow at 75% of forced vital capacity predicted; FEF25–75%pred, forced expiratory flow at 25–75% of forced vital capacity predicted.

**Table 2:** Descriptive statistics of ambient environmental variables at lag0 (*n* = 399).

Variables	Mean (SD)	Median (IQR)	Min	Max
O_3_ (μg/m^3^) ^1^	111.01 (45.01)	102.35 (63.97)	18.40	244.10
Warm season	129.72 (38.65)	125.75 (63.57)	67.60	244.10
Cold season	70.64 (28.14)	70.15 (28.47)	18.40	165.60
PM_2.5_ (μg/m^3^) ^2^	37.49 (30.07)	29.60 (33.90)	1.00	168.40
Warm season	28.42 (20.23)	24.30 (25.10)	1.00	140.60
Cold season	57.02 (37.64)	49.25 (57.60)	3.70	168.40
BC ^3^	1.44 (1.13)	1.11 (1.23)	0.04	6.35
OM ^4^	7.83 (5.96)	6.23 (6.93)	0.27	37.56
SO_4_^2−^	4.78 (4.33)	3.56 (4.59)	0.13	30.50
NO_3_^−^	6.83 (7.66)	4.30 (6.34)	0.12	42.99
NH_4_^+^	4.15 (4.53)	2.61 (3.85)	0.09	25.28
Temperature (K)	288.71 (9.54)	291.78 (15.36)	258.30	307.09
Relative humidity (%)	51.54 (19.12)	50.56 (31.59)	14.57	94.42

^1^ O_3_, ozone; ^2^ PM_2.5_, fine particulate matter; ^3^ BC, black carbon; ^4^ OM, organic matter.

## Data Availability

The data supporting the findings of this study are available from the corresponding author upon reasonable request.

## Supplementary Materials

The following supporting information can be downloaded at https://www.mdpi.com/article/10.3390/toxics13040279/s1, Figure S1: Associations between small airway function parameters and ambient PM_2.5_ at lag0–lag7; Figure S2: Effects of ambient O_3_ at lag0–lag7 on small airway function parameters across different temperature levels; Figure S3: Effects of ambient O_3_ at lag0–lag7 on small airway function parameters across warm and cold seasons; Figure S4: Stratified analyses of the associations between ambient O_3_ at lag0–lag7 and small airway function parameters on the basis of sex; Figure S5: Stratified analyses of the associations between ambient O_3_ at lag0–lag7 and small airway function parameters on the basis of the allergic asthma phenotype; Figure S6: Stratified analyses of the associations between ambient O_3_ at lag0–lag7 and small airway function parameters on the basis of medication use; Figure S7: Associations between small airway function parameters and ambient O_3_ at lag0–lag7 (Incorporating PM_2.5_ as an additional control variable); Figure S8: Associations between small airway function parameters and ambient O_3_ at lag0–lag7 (Controlling for lag0–7 mean temperature and relative humidity).

## Abbreviations

The following abbreviations are used in this manuscript:

BMI	body mass index
BC	black carbon
FEF	forced expiratory flow
FVC	forced vital capacity
FEF50%	forced expiratory flow at 50% of forced vital capacity
FEF75%	forced expiratory flow at 75% of forced vital capacity
FEF25–75%	forced expiratory flow at 25–75% of forced vital capacity
FEV1	forced expiratory volume in one second
NOx	nitrogen oxides
NO_3_^−^	nitrate
NH_4_^+^	ammonium
O_3_	ozone
OM	organic matter
PM_2.5_	fine particulate matter
Pre-BD	prebronchodilator
Post-BD	postbronchodilator
RH	relative humidity
SO_4_^2−^	sulfate
SAD	small airway dysfunction
T	temperature
VOCs	volatile organic compounds
