# Supplementary material for: Short-Term Effect of Ozone Exposure on Small Airway Function in Adult Asthma Patients with PM2.5 Exacerbating the Effect
Source: Toxics. 2025 Apr 5;13(4):279. doi: 10.3390/toxics13040279 (PMC12031310; doi:10.3390/toxics13040279)
Supplement: Supplementary file 1 [file toxics-13-00279-s001.zip › toxics-3553431-supplementary.pdf]

Supplementary materials

Short-Term Effect of Ozone Exposure on Small Airway Function in Adult Asthma Patients with $PM_{2.5}$ Exacerbating the Effect

Authors

Ying Shang [1,2,†], Yanjing Liang [1,2,†], Dongxia Jiang [3], Zhengxiong Li [3], Xianlin Mu [4], Xuehu Han [5], Xinzhuo Xie [5], Guanglong Fu [5], Yunshu Zhang [5], Yongchang Sun [1,2], Shaodan Huang [3,6,*] and Chun Chang [1,2,*]

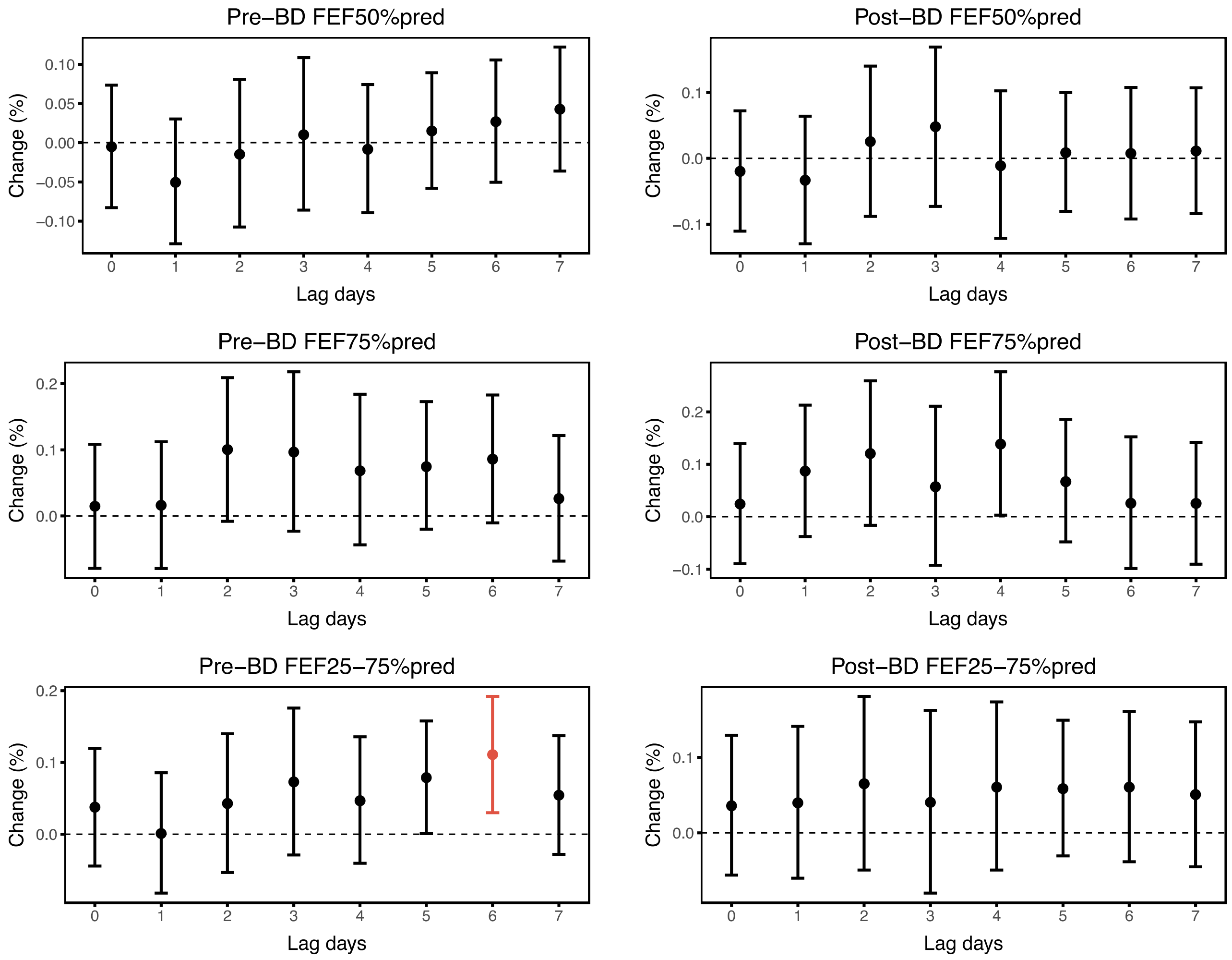

Figure S1. Associations between small airway function parameters and ambient $PM_{2.5}$ at lag0-lag7. The red line indicates $p < 0.05$.

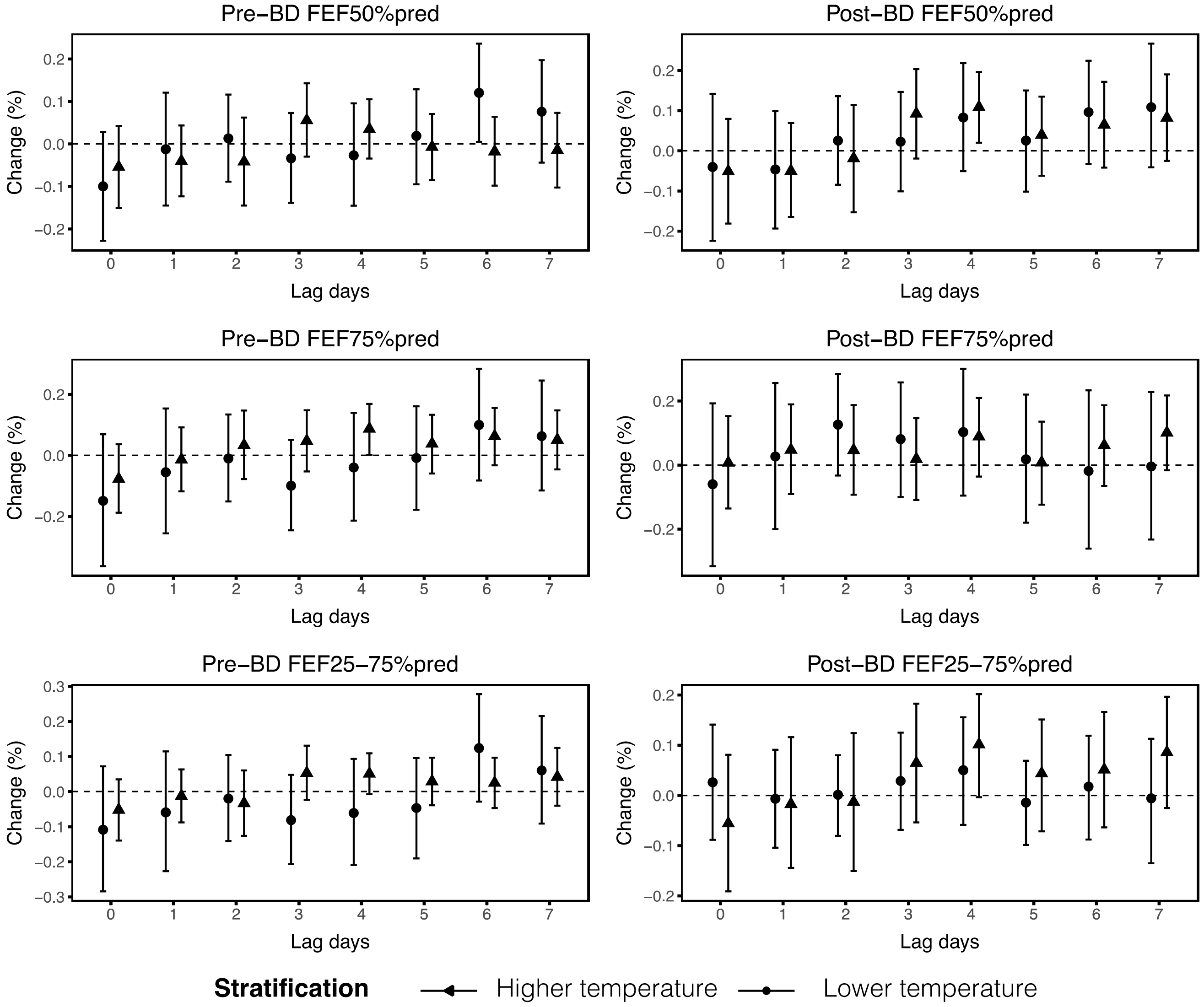

Figure S2. Effects of ambient O_3 at lag0-lag7 on small airway function parameters across different temperature levels.

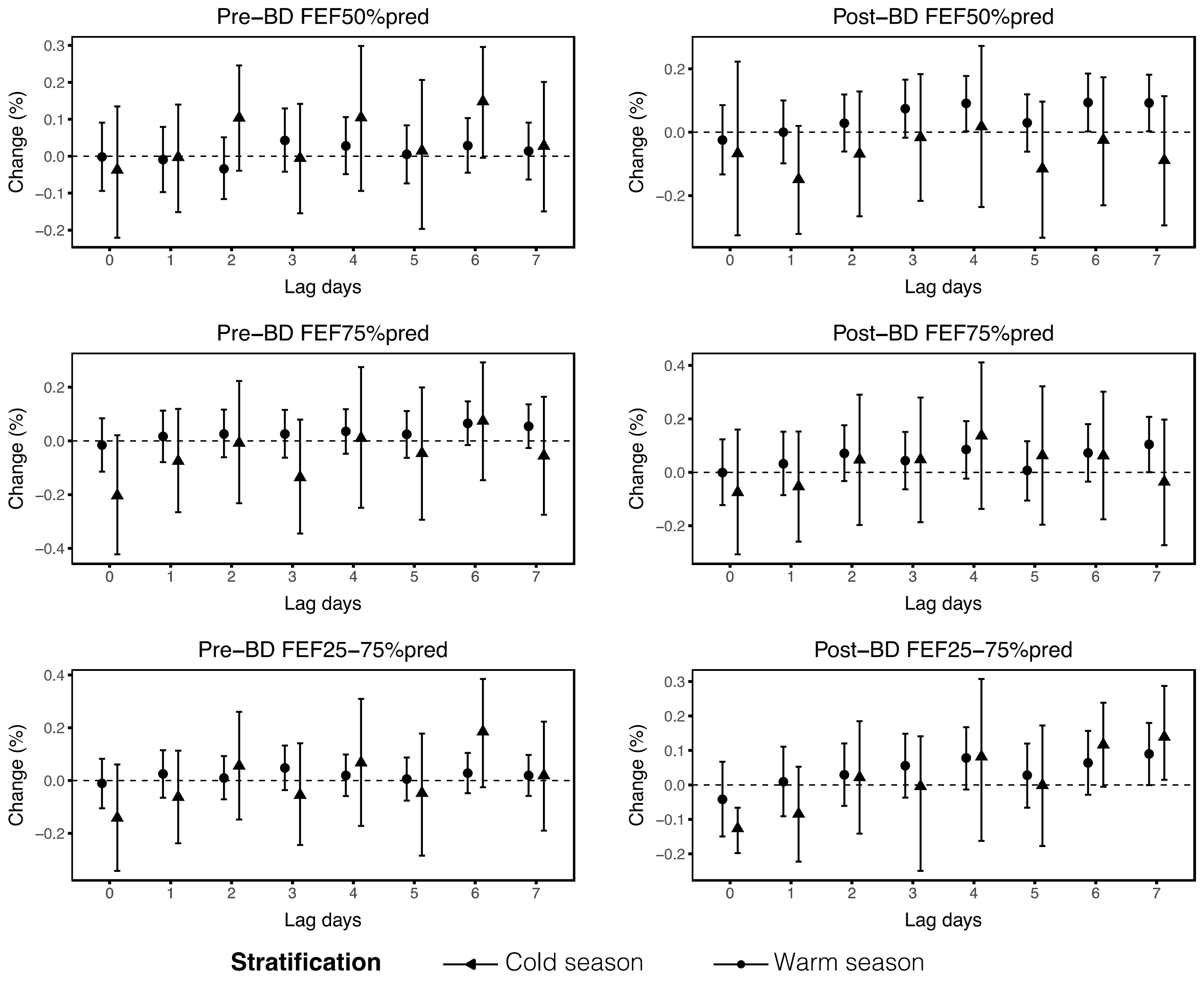

Figure S3. Effects of ambient O_3 at lag0-lag7 on small airway function parameters across warm and cold seasons.

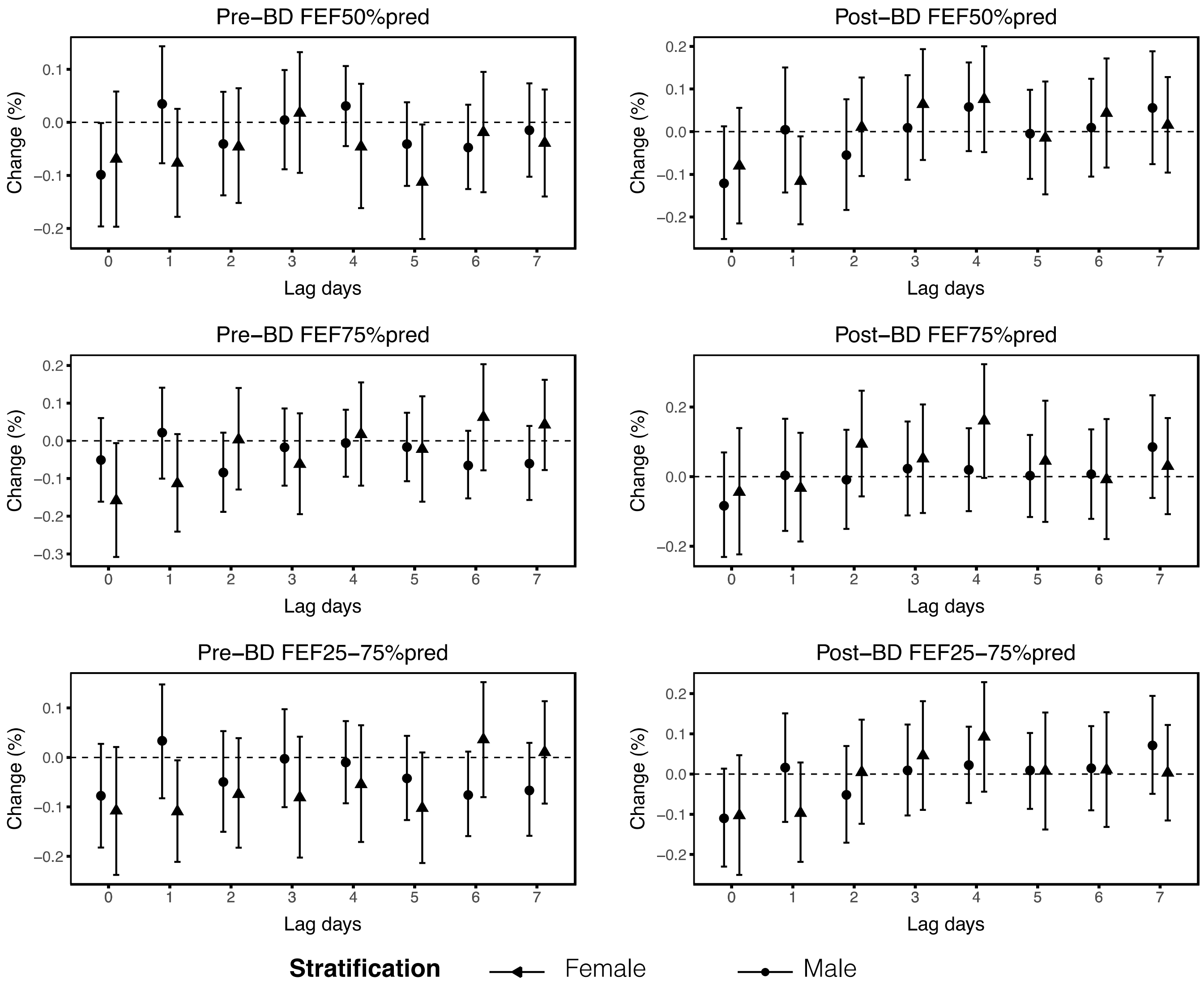

Figure S4. Stratified analyses of the associations between ambient O_3 at lag0-lag7 and small airway function parameters on the basis of sex.

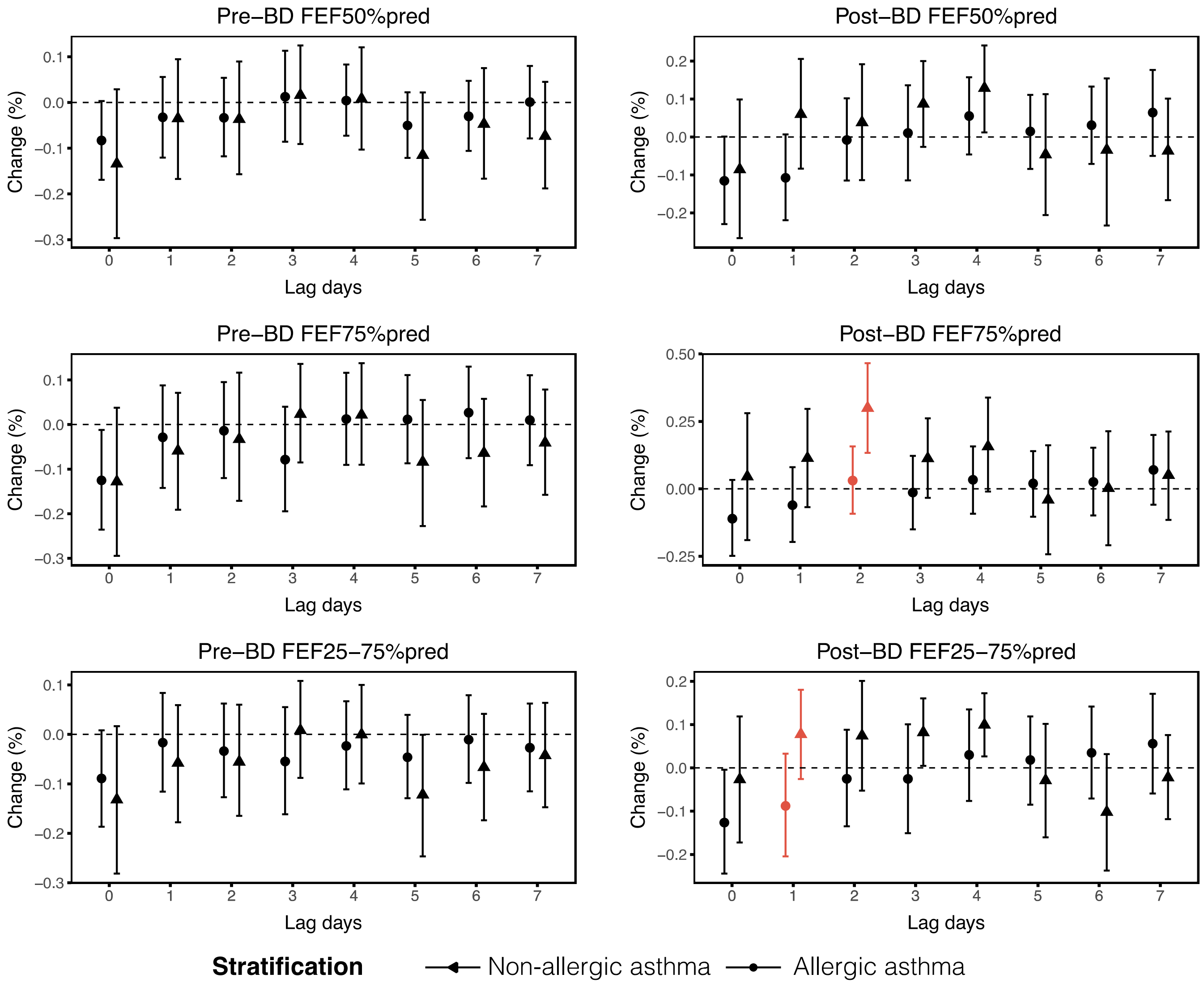

Figure S5. Stratified analyses of the associations between ambient O_3 at lag0-lag7 and small airway function parameters on the basis of the allergic asthma phenotype. The red lines indicate significant differences in the associations between O_3 and small airway function parameters in allergic asthma and non-allergic asthma ($p < 0.05$).

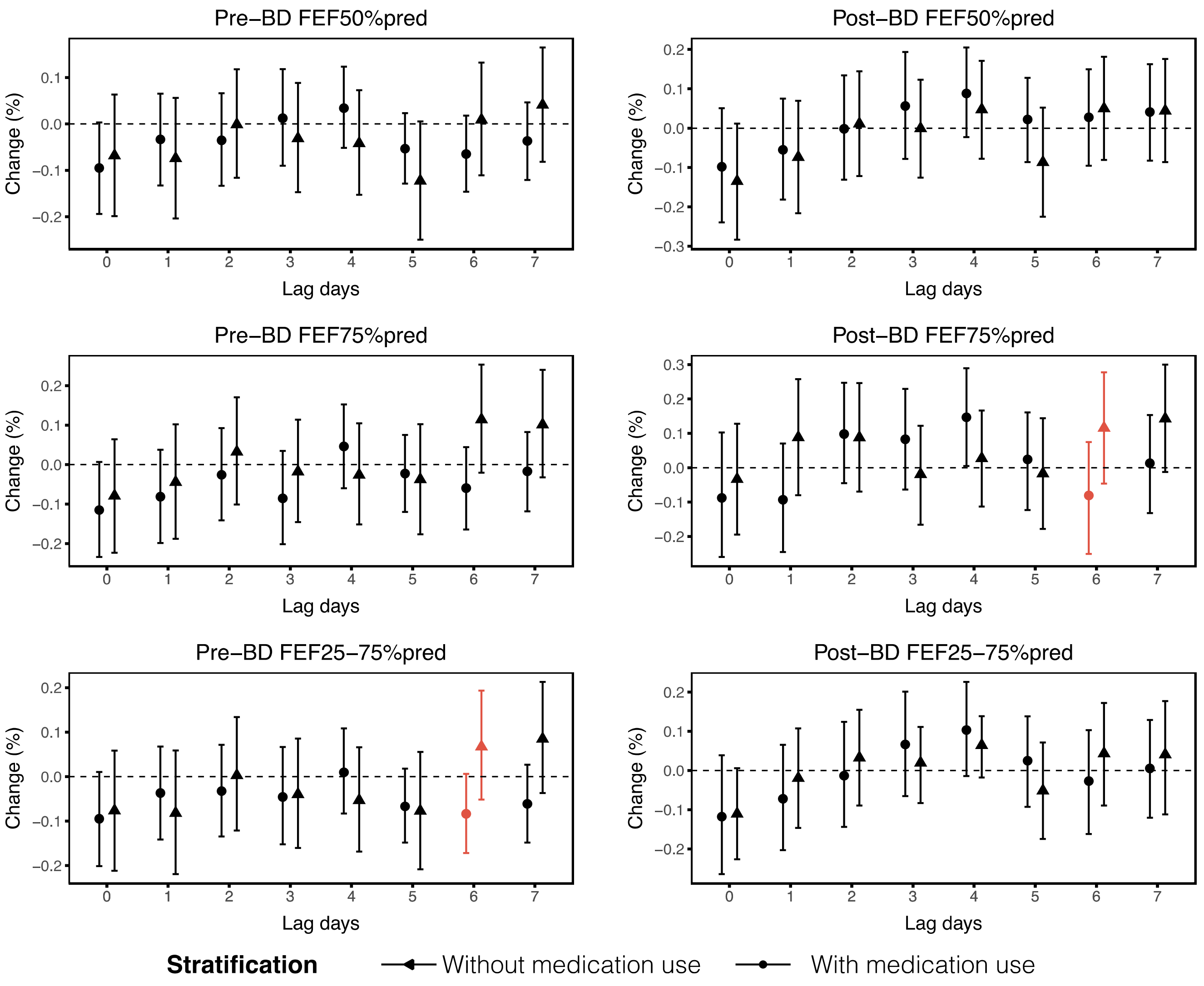

Figure S6. Stratified analyses of the associations between ambient O_3 at lag0-lag7 and small airway function parameters on the basis of medication use. The red lines indicate significant differences in the associations between O_3 and small airway function parameters in individuals with and without medication use.

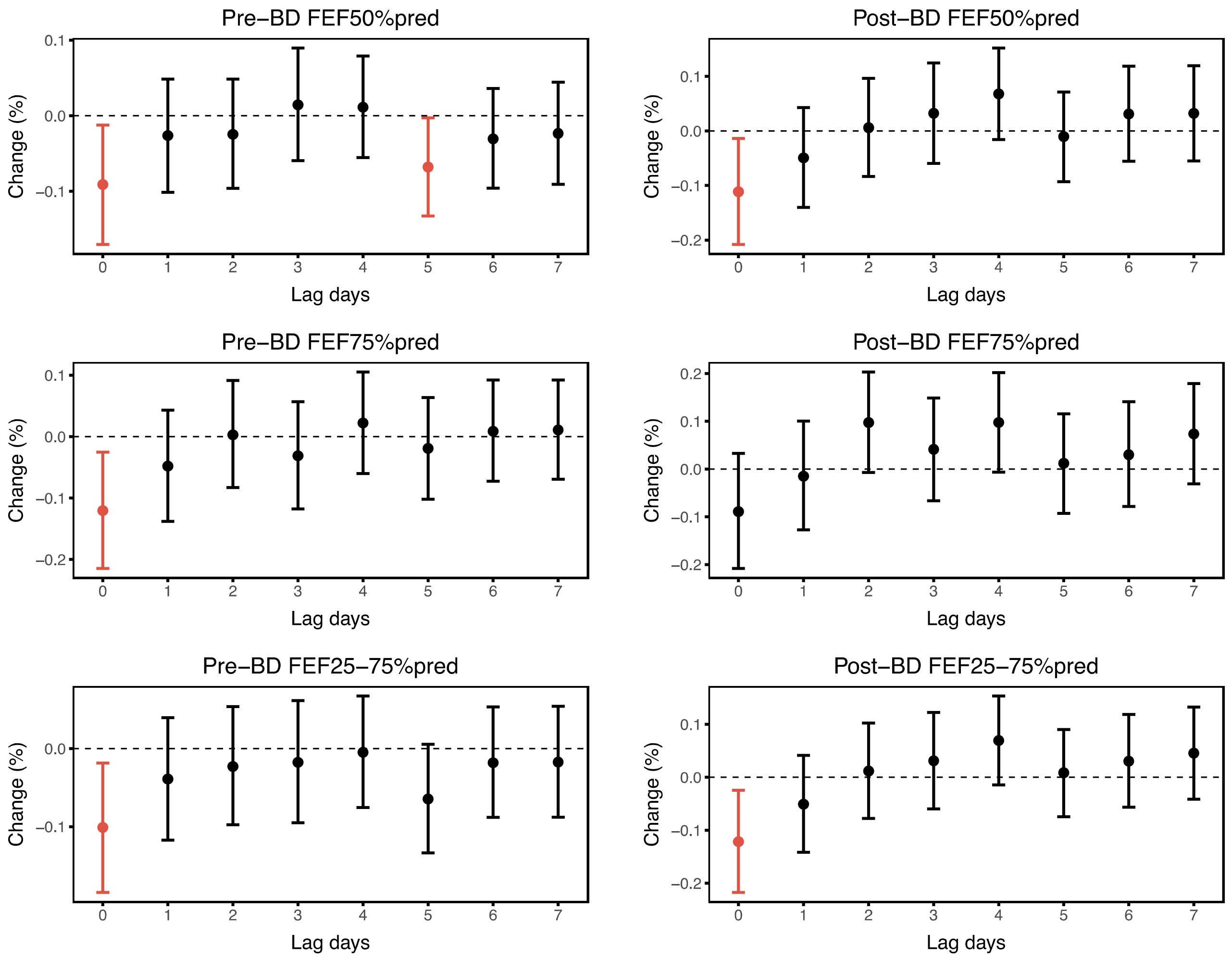

Figure S7. Associations between small airway function parameters and ambient O_3 at lag0-lag7 (Incorporating $PM_{2.5}$ as an additional control variable). The red lines indicate $p < 0.05$.

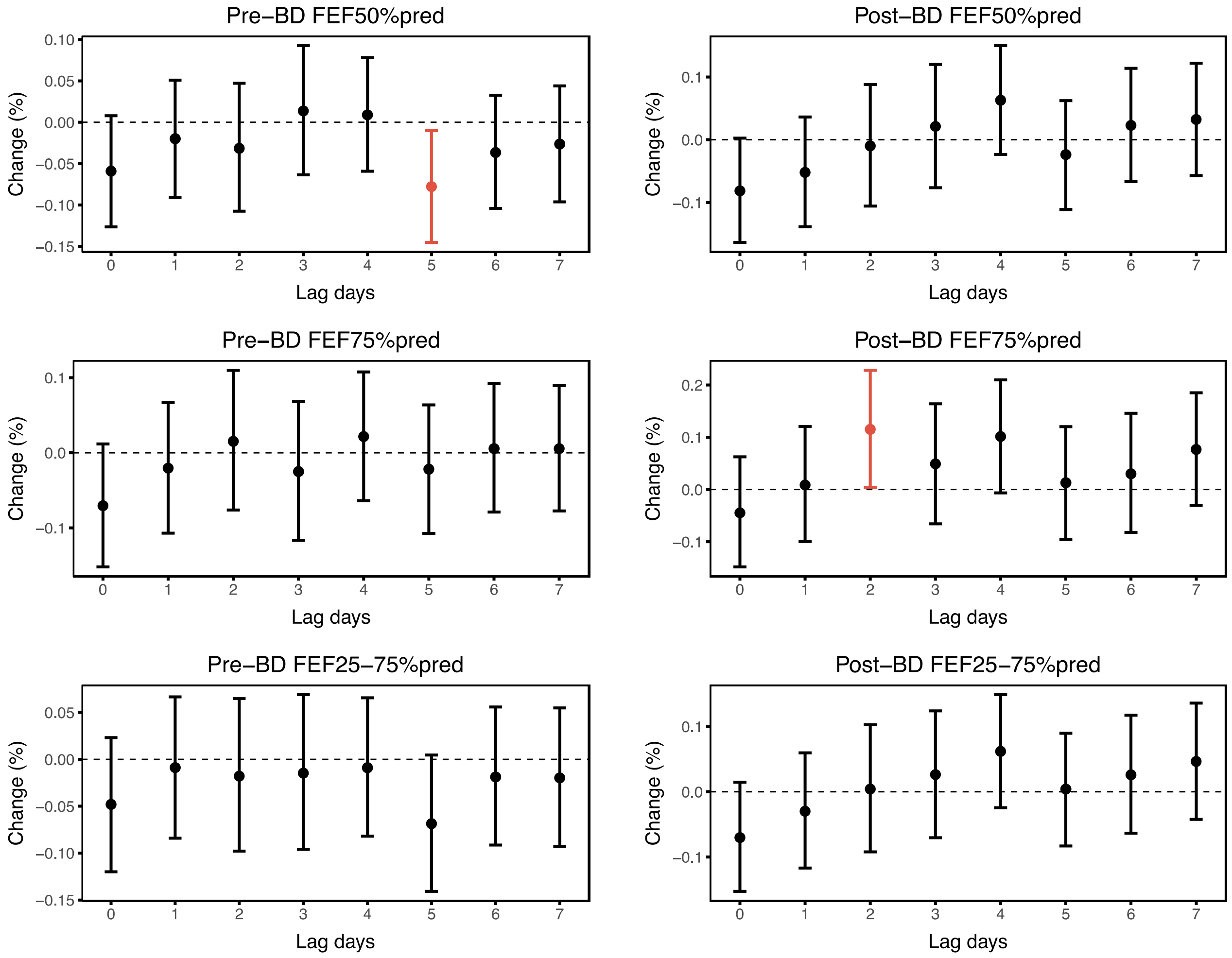

Figure S8. Associations between small airway function parameters and ambient O_3 at lag0-lag7 (Controlling for lag0-7 mean temperature and relative humidity). The red lines indicate $p < 0.05$.
